# Establishment and characterization of mouse lymph node fibrosis models

**DOI:** 10.1002/ame2.70261

**Published:** 2026-08-03

**Authors:** Yaru Niu, Xu Guan, Jian Ma, Xin Zhang, Yihang Shi, Jinzhu Zhang, Leiqiong Gao, Wei Chen, Xishan Wang

**Affiliations:** ^1^ Department of Colorectal Surgery, National Cancer Center, National Clinical Research Center for Cancer, Cancer Hospital Chinese Academy of Medical Sciences and Peking Union Medical College Beijing China; ^2^ Department of Surgical Oncology and General Surgery First Hospital of China Medical University Shenyang China; ^3^ Institute of Immunological Innovation and Translation Chongqing Medical University Chongqing China; ^4^ Beijing Clinical Research Institute, Beijing Friendship Hospital Capital Medical University Beijing China

**Keywords:** extracellular matrix, fibrosis, lymph node, mouse model, transforming growth factor‐β1

## Abstract

**Background:**

Lymph node (LN) fibrosis occurs in a variety of pathological conditions, including HIV infection, obesity, cancer, and tissue injury. Fibroblastic reticular cells (FRCs) play a critical role in maintaining LN architecture and immune homeostasis, whereas their dysregulation promotes extracellular matrix (ECM) deposition and immune dysfunction. Transforming growth factor‐β1 (TGF‐β1) is a key profibrotic cytokine. However, reliable and convenient animal models for investigating LN fibrosis remain limited.

**Methods:**

This study comprehensively compared four induction methods: footpad injection of TGF‐β1, lymphatic vessel ligation, inguinal subcutaneous injection of TGF‐β1, and direct intra‐LN injection of TGF‐β1. Histological analysis, transcriptomic profiling, flow cytometric analysis, and safety evaluation were performed to assess fibrosis and immune alterations.

**Results:**

Among the four approaches, inguinal subcutaneous and intra‐LN injection of TGF‐β1 successfully induced LN fibrosis without causing significant injury to major organs. Notably, the intra‐LN injection model induced fibrosis in both cortical and medullary regions of LNs. Comparing the transcriptomic data of fibrotic and non‐fibrotic LNs demonstrated marked changes in fibrosis‐related genes, including pro‐fibrogenic mediators, collagens and basement membrane‐related genes, and TGF‐β1‐associated signaling pathways. In two fibrotic models, we also noticed increased macrophage infiltration and a drop in CD8^+^ T cells, suggesting an immune suppressive microenvironment. Furthermore, inhibition of collagen cross‐linking partially alleviated fibrotic remodeling in fibrotic LNs.

**Conclusion:**

We established and characterized two mouse models of LN fibrosis induced by TGF‐β1 administration. These models provide valuable tools for investigating the mechanisms of LN fibrosis and its impact on local immune regulation, and may facilitate the development of therapeutic strategies targeting fibrotic LNs.

## Graphical abstract

The design of the entire paper. Schematic illustration of four strategies for establishing mouse lymph node (LN) fibrosis models. The study further compares their fibrotic remodeling patterns and immune alterations.

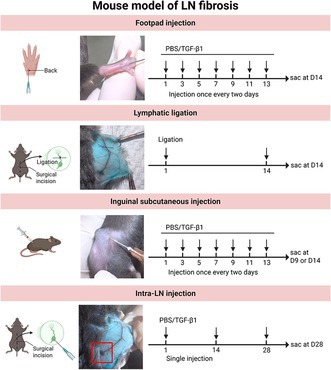

## 1 INTRODUCTION

Lymph nodes (LNs) are the initial sites for activating adaptive immune responses, and their three‐dimensional network structure is composed of fibroblast reticulum cells (FRCs) and extracellular matrix (ECM) channels.^1^ This conduit network supports the survival of T cells, B cells, dendritic cells, plasma cells, and macrophages. FRCs funnel antigens and antigen‐presenting cells to rare antigen‐specific lymphocytes and control matrix production during the immune response.^2^ Chronic inflammation, as it occurs in cancers or human immunodeficiency virus (HIV) infections, leads to the abnormal accumulation of ECM components, resulting in LN fibrosis. LN fibrosis has been described previously in renal ischemia–reperfusion injury^3^ or unilateral ureteral obstruction.^4^ The FRC response to tissue repair drives increased collagen synthesis, ultimately leading to fibrosis.

Transforming growth factor‐β1 (TGF‐β1) is a critical mediator of crosstalk between FRC activation and LN fibrosis. The signaling in FRCs contributes to the progression of fibrosis.^5^, ^6^ Secreted collagen accumulates around FRCs separate immune cells from them, so the naive lymphocytes die as they lose complete contact with FRCs. In HIV infection, high expression of TGF‐β1 in CD8^+^ T cell has been associated with increased type I collagen deposition, persistent immune activation in the fibrotic LNs.^7^ Moreover, LN fibrosis may impair vaccine efficacy. Previous studies have shown that fibrosis in LNs from individuals receiving the yellow fever vaccination was associated with reduced T‐cell numbers and weakened humoral immune responses, suggesting that higher baseline immune activation in certain geographic regions may contribute to LN fibrosis and impaired vaccine responses.^8^ Persistent, low‐grade inflammatory state caused by obesity can weaken immune function. The T‐cell apoptosis is previously assumed to occur as a result of excessive visceral adiposity.^9^ During organ transplantation, senescent FRCs produce large amounts of type I collagen and undergo phenotypic transformation toward a myofibroblast.^10^ According to one study, patients with a higher proportion of fibrotic metastatic LNs tend to have worse survival.^11^ Our recent research has identified a subtype of tumor‐draining LNs without detectable metastasis but showing clear fibrotic traits, along with fewer T and B cells and a marked reduction in follicular dendritic cells.^12^

Previous studies have established various animal models for studying fibrosis in vital organs. Organ fibrosis has been achieved using various methods, such as induction of pulmonary fibrosis through silica exposure,^13^ combined bleomycin and lipopolysaccharide exposure,^14^ liver fibrosis induced by bile duct ligation,^15^ and myocardial fibrosis induced by *MYBPC3* gene knockout.^16^ Moreover, although there are some progressions, no systematic in vivo model has been established yet. Developing a simple and reliable way to model LN fibrosis is urgently needed to support mechanistic research and the effective therapies.

In this study, we tested four approaches to induce LN fibrosis. The methods of subcutaneous and intra‐LN injection of TGF‐β1 were able to produce fibrotic changes in the LNs. Neither method caused liver or kidney damage, and routine blood tests appeared normal. Transcriptomic analysis explored the pathological features and underlying mechanisms of the two LN fibrosis models. Moreover, fibrotic LNs exhibited altered immune cell composition, characterized by reduced proportions of naive and memory CD8^+^ T cells together with increased infiltration of both M1‐ and M2‐like macrophages.

## 2 MATERIALS AND METHODS

### 2.1 Mouse experiments

Eight‐week‐old male C57BL/6J mice (weighing 22–25 g) were purchased from Vital River Laboratory Animal Technology Co., Ltd. (Beijing, China). All mice were housed in a standardized environment with a 12‐h light/dark cycle and free access to standard feed and sterile drinking water. To establish a robust model of LN fibrosis, we tested four intervention regimens using C57BL/6J mice. The method included footpad injection, lymphatic vessel ligation, inguinal subcutaneous injection, and intra‐LN injection. Recombinant human TGF‐β1 protein (PEPROTECH, New Jersey, USA), which has been widely used to induce fibrosis in mouse models and cells,^17^, ^18^ was used in the experiments.

In the footpad injection method, TGF‐β1 (0.5 μg/50 μL) or phosphate‐buffered saline (PBS) was administered into the footpad every 2 days for 2 weeks. As previously reported, tracer dye (1% w/v Evans Blue) was injected subcutaneously and allowed to drain to the inguinal LNs and lymphatic vessels to enable visualization.^19^ In the lymphatic vessel ligation group, efferent lymphatic vessels were surgically ligated to block lymphatic drainage. In the intra‐LN injection method, the skin was carefully incised to expose the inguinal LN, and TGF‐β1 (0.2 μg/μL) or PBS was directly injected into the inguinal LN using an insulin needle at a total volume of 10 μL per mouse. Samples were collected at 2 or 4 weeks postinjection. For the inguinal subcutaneous injection group, TGF‐β1 (0.01 μg/μL) or PBS was injected subcutaneously around the inguinal LN every 2 days for 1 to 2 weeks, with a total injection volume of 50 μL per mouse. The TGF‐β1 dosage used in this study was determined based on previously reported fibrosis models that successfully induced local fibrotic changes in vivo.^20^

To evaluate the reversibility of LN fibrosis, a lysyl oxidase inhibitor (LOXi), β‐aminopropionitrile (BAPN; Sigma–Aldrich, St. Louis, MO, USA), was used for antifibrotic treatment. LN fibrosis was induced by intra‐LN injection of TGF‐β1, as described above. Mice in the antifibrotic group received drinking water containing the LOXi (~3 mg/kg per day), whereas control mice received normal drinking water. After the end of the treatment, euthanasia of the group of mice was carried out by cervical dislocation, and the serum was collected. The collected inguinal LNs were fixed with 4% paraformaldehyde (PFA) for histological analysis, and the rest were immediately frozen in liquid nitrogen for further molecular experiments. All animal procedures were approved by the Animal Control Committee of the National Cancer Center/National Clinical Research Center for Cancer/Cancer Hospital of the Chinese Academy of Medical Sciences and Peking Union Medical College (NCC2025A416).

### 2.2 Hematoxylin–eosin staining

LN, heart, liver, spleen, lung, and kidney tissues were immediately fixed in 4% PFA, embedded in paraffin, and sectioned (5 μm thick). Samples were stained with hematoxylin–eosin (HE), and LN sections were also stained with Sirius red. The specific procedures were performed according to the manufacturer's protocols.

### 2.3 Immunohistochemistry

Formalin‐fixed and paraffin‐embedded tissue sections were dewaxed and hydrated, and antigen retrieval was performed in citrate buffer (pH 6.0). Incubation with 3% H_2_O_2_ solution for 15 min was performed to block endogenous peroxidase activity. Sections were incubated overnight at 4°C with the following primary antibodies: α‐smooth muscle actin (α‐SMA) (CST, Danvers, MA, USA, #19245, 1:100), collagen I (Absin, Shanghai, China, #120555, 1:200), CD45 antibody (Proteintech, Wuhan, China, 20103‐1‐AP, 1:200), CD8a monoclonal antibody (Proteintech, Wuhan, China, 66868‐1‐IG, 1:200), and anti‐CD68 antibody (Abcam, Cambridge, UK, ab303565, 1:200). After PBS washes, the slides were incubated with horseradish peroxidase (HRP)‐conjugated secondary antibody at room temperature for 1 h, followed by 3,3′‐diaminobenzidine (DAB) 4′,6‐diamidino‐2‐phenylindole staining and hematoxylin counterstaining. Images were acquired using an optical microscope, and six fields of view were randomly selected from each slice. The proportion of positively stained areas was analyzed using ImageJ software.

### 2.4 Immunofluorescence

Paraffin‐embedded LN tissues were sectioned at 4 μm thickness. Sections were fixed with cold acetone for 5 min, then washed with PBS and blocked with 3% bovine serum albumin (BSA) to reduce nonspecific binding. The sections were then incubated overnight at 4°C with following primary antibodies specific for the indicated mouse antigens—rabbit recombinant monoclonal antibody PDPN (Abcam, Cambridge, UK, AB319138, 1:200), rabbit recombinant monoclonal antibody α‐SMA (Proteintech, Wuhan, China, 80008‐1‐RR, 1:200), rabbit monoclonal antibody iNOS (CST, Danvers, MA, USA, #13120, 1:200), and rabbit monoclonal antibody CD206 (CST, Danvers, MA, USA, #91992, 1:200). The sections were then exposed to the corresponding fluorophore‐conjugated secondary antibodies at a dilution of 1:500. 4′,6‐diamidino‐2‐phenylindole (DAPI) (VECTASHIELD, Vector Laboratories,Newark, CA, USA) was used to stain the nuclei. Fluorescence images were acquired using immunofluorescence microscopy. Quantitative analyses were performed using ImageJ software.

### 2.5 Flow cytometry

Freshly isolated mouse LNs were mechanically dissociated into single‐cell suspensions through a 70‐μm cell strainer in cold PBS. Cells were stained with fixable viability dye live/dead violet (LIVE/DEAD Violet, #L34958, Thermo Fisher Scientific, Waltham, MA, USA) and combinations of the following fluorophore‐conjugated antibodies: anti‐mouse CD8 (clone#53‐6.7), CD62L (clone#MEL‐14), CD44 (clone#IM7; all Biolegend, San Diego, CA, USA). All flow cytometry was performed on LSR Fortessa (BD Biosciences, San Jose, CA, USA) analyzers, and offline analysis was performed using FlowJo software (Treestar, version 10.7.2).

### 2.6 Measurement of biochemical indicators

Mice were fasted for 12 h after the last administration, and whole blood samples were collected via the retro‐orbital bleeding route. Samples were centrifuged at 3000 rpm for 15 min, and the supernatant serum was stored at −80°C for subsequent use. We determined the levels of aspartate aminotransferase (AST), alanine aminotransferase (ALT), blood urea nitrogen (BUN), and creatinine (CRE) according to the manufacturers' instructions. The absorbance of each well was measured at a specific wavelength. Standard curves were plotted using the corresponding calculation formulas to calculate the final concentrations of AST, ALT, BUN, and CRE.

### 2.7 RNA sequencing

Tissue samples were collected and stored at −80°C for RNA sequencing. Total RNA was isolated from tissue frozen in liquid nitrogen, and its quality was assessed using the Bioanalyzer (Agilent Technologies, Santa Clara, CA, USA). RNA integrity number (RIN) > 6.0 was considered acceptable for library construction. After ribosomal RNA was removed using the Ribo‐Zero rRNA Removal Kit (Epicenter, Madison, WI, USA), sequencing libraries were prepared using the Illumina TruSeq RNA Sample Preparation Kit (Illumina,San Diego, CA, USA). The complementary DNA (cDNA) libraries were quantified using the Qubit dsDNA HS Assay Kit and a Qubit 3.0 fluorometer (Thermo Fisher Scientific, Waltham, MA, USA). Paired‐end 50‐bp sequencing was performed on the Illumina HiSeq2500 platform, with two samples allocated per lane. Raw reads were aligned to the mouse reference genome (mm10) using STAR (version 2.5.2b), and gene expression levels were normalized as fragments per kilobase of transcript per million mapped reads (FPKM) values for subsequent analysis. RNA‐seq data have been deposited in the GEO database under accession number GSE330392.

### 2.8 Differentially expressed gene analysis

Differentially expressed genes (DEGs) between fibrotic and nonfibrotic LNs were identified using the *DESeq* R package. Genes with adj.*p*‐value <0.05 and |log2 fold change| > 1 were defined as significantly upregulated or downregulated genes, respectively. Volcano plots were generated to visualize DEGs, and gene expression heatmaps were generated using the *pheatmap* R package based on *z*‐score normalized FPKM values.

### 2.9 Real‐time PCR

Total RNA was isolated from mouse tissues, and equal amounts (1 μg) of RNA from each sample were reverse transcribed according to the instructions of the reverse transcription polymerase chain reaction (RT‐PCR) Transcriptor First Strand cDNA Synthesis Kit. Quantitative real‐time PCR was performed with Fast SYBR Green Master Mix on a QuantStudio real‐time PCR system. *Gapdh* was used as an endogenous reference gene, and the relative expression level of the target gene was calculated using the 2^−ΔΔCt^ method. The primer sequences used were as follows: *Gapdh*: forward 5′‐AGCCACATCGCTCAGACAC‐3′, reverse 5′‐AGGCAGGTTTGATCTCCGTT‐3′; *Col1a1*: forward 5′‐CCTGGTAAAGATGGTGCC‐3′, reverse 5′‐CACCAGGTTCACCTTTCGCACC‐3′; *Acta2*: forward 5′‐CTGACAGAGGCACCACTGAA‐3′, reverse 5′‐CATCTCCAGAGTCCAGCACA‐3′.

### 2.10 Evaluation of immune cell composition

Immune cell composition in fibrotic and nonfibrotic LNs was estimated using ImmuCC, a mouse‐specific immune cell deconvolution algorithm based on transcriptomic profiles. A reference signature matrix (“mice”) was used for cell‐type deconvolution. Relative proportions of immune cell subsets in each sample were estimated from bulk RNA‐seq expression data using a support vector regression‐based deconvolution algorithm based on gene expression signatures.

### 2.11 Statistical analysis

Data are displayed as means ± standard error of the mean (SEM). Statistical significance between two groups of continuous variables was analyzed using an unpaired two‐tailed Student's *t*‐test, and a *p*‐value <0.05 was considered statistically significant. Statistical significance was performed using GraphPad Prism software.

## 3 RESULTS

### 3.1 Overview of four strategies for establishing LN fibrosis models

**FIGURE 1:**
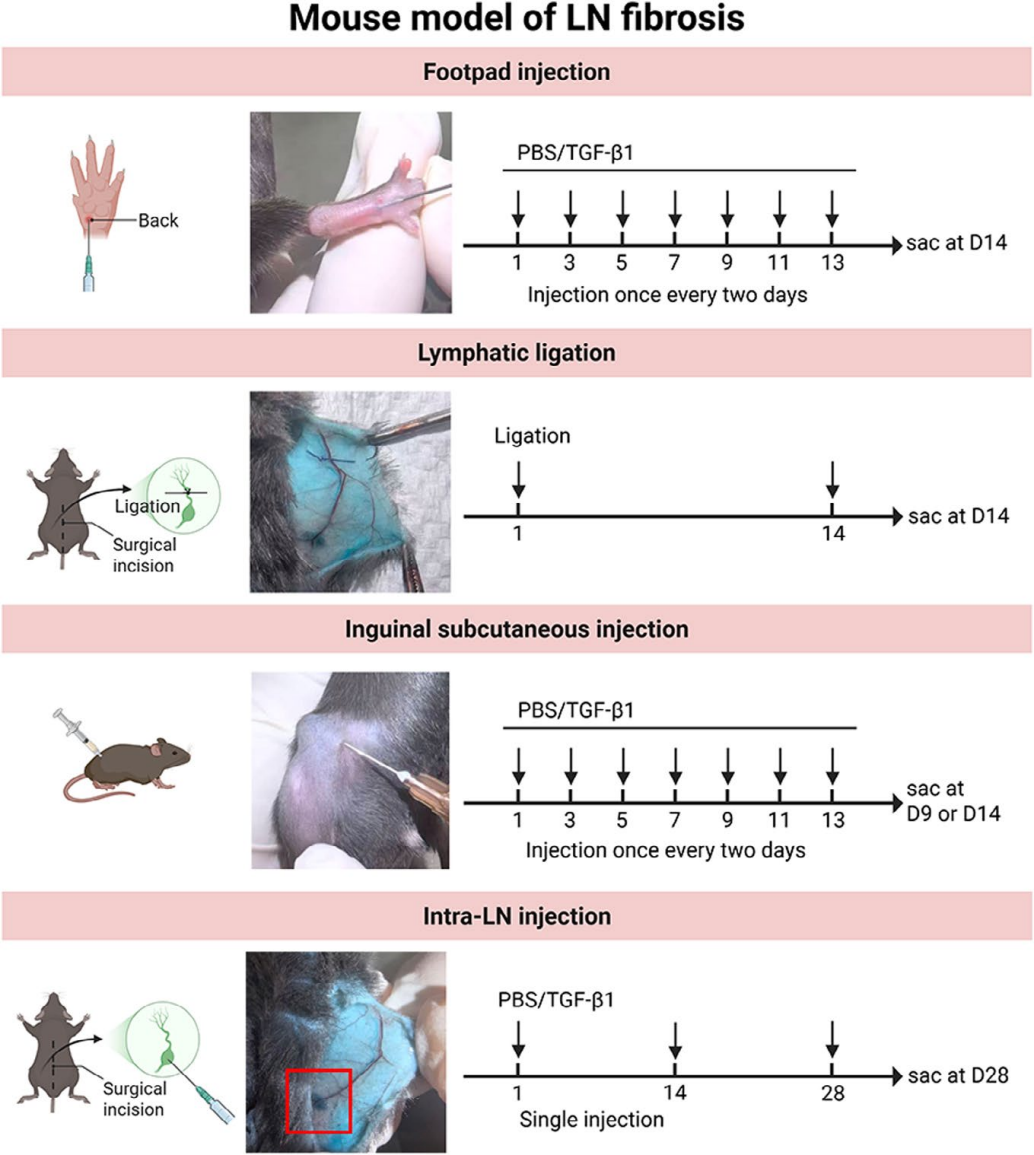
Schematic illustration of four experimental strategies for inducing lymph node (LN) fibrosis. Strategy 1 (footpad injection): transforming growth factor‐β1 (TGF‐β1) was injected subcutaneously through the footpad of the hind limb, draining to the inguinal lymph nodes via afferent lymphatic vessels. Strategy 2 (lymphatic vessel ligation): after subcutaneous injection of 1% w/v Evans Blue dye to visualize the lymphatic vessels, the efferent lymphatic vessel distal to the inguinal LN was surgically ligated. Strategy 3 (inguinal subcutaneous injection): direct subcutaneous injection of TGF‐β1 into the area adjacent to the inguinal LNs. Strategy 4 (intra‐LN injection): 1% w/v Evans Blue dye was injected to visualize the inguinal LN and associated lymphatic vessels. The LN was then surgically exposed through a small incision, followed by direct injection of TGF‐β1 into the inguinal LN.

To establish reproducible LN fibrosis mouse model, we examined four different induction strategies, namely footpad injection of TGF‐β1, lymphatic vessel ligation, inguinal subcutaneous injection of TGF‐β1, and intra‐LN injection of TGF‐β1. The injection and surgical sites are illustrated in Figure 1. At the endpoint, there was no significant difference in body and LN weights (Figure S1A,B). Next, we optimized the duration of TGF‐β1 exposure. Sirius red staining revealed that subcutaneous injection of TGF‐β1 for 1 week and intra‐LN injection of TGF‐β1 for 2 weeks induced mild collagen deposition (Figure S1C). CD45 immunohistochemistry analysis showed expansion of CD45^+^ cells (Figure S1D). After prolonged treatments, the injected treatments both produced progressively more collagen and immune cells. Thus, subcutaneous injection of TGF‐β1 for 2 weeks and intra‐LN injection of TGF‐β1 for 4 weeks were the optimal modeling duration (Figure S1C,D).

### 3.2 Increased expression of fibrotic and inflammatory biomarkers in LN fibrosis models

**FIGURE 2:**
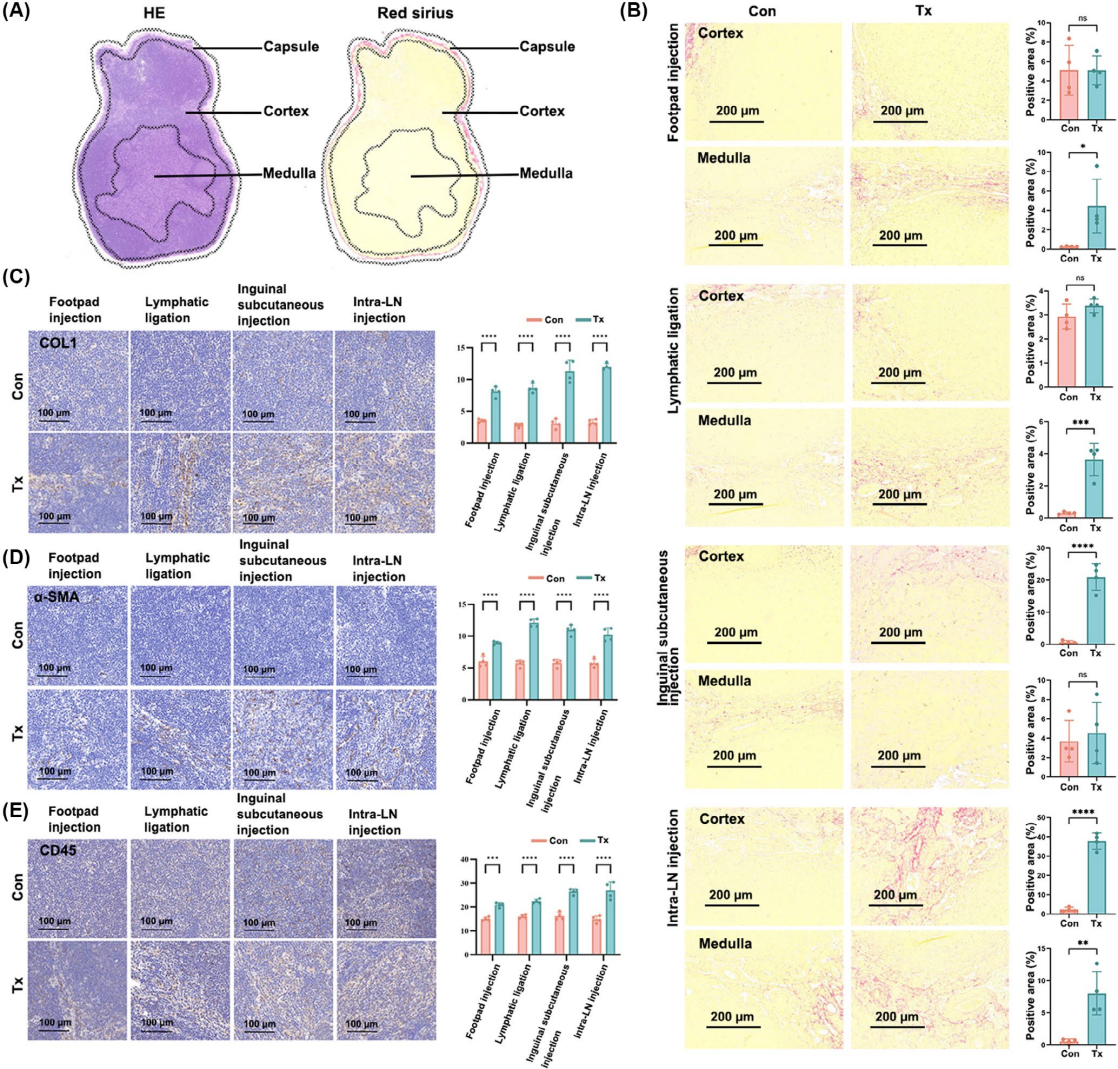
Evaluation of lymph node (LN) fibrosis and inflammatory responses induced by four modeling strategies. Histological analysis of LNs following four fibrosis induction strategies. (A) Representative hematoxylin–eosin (HE) and Sirius red staining images illustrating the anatomical regions of the LN, including the capsule, cortex, and medulla. Dashed lines indicate the boundaries of each region. (B) Representative Sirius red staining and regional quantification of collagen deposition in the cortex and medulla of LNs across four fibrosis‐induction models and corresponding control groups (scale bars: 200 μm, *n* = 6 random microscopic fields of sections from four mice). (C‐E) Representative immunohistochemical staining and quantification of COL1, α‐smooth muscle Actin (α‐SMA), and CD45 in control and fibrotic LNs (scale bars: 100 μm, *n* = 6 random microscopic fields of sections from four mice). Data are presented as mean ± standard error of the mean (SEM). *p < 0.05, **p < 0.01, ***p < 0.001, ****p < 0.0001; ns, not significant.

To evaluate the extent and regional distribution of LN fibrosis, full LN sections from all four experimental groups were analyzed by Sirius red staining. All four methods induced collagen deposition in the LNs, but the regional patterns and pathological features were much different (Figure S2A). To further define the fibrotic regions within the LNs, LN anatomical structures were identified based on HE staining. As shown by HE staining, the LN capsule appeared as a thin outer connective tissue layer. The cortex was characterized by densely packed lymphocytes beneath the capsule, whereas the medulla showed relatively loose cellular organization with visible medullary cords and sinus‐like structures (Figure S2B). According to the corresponding regions identified in adjacent HE‐stained sections, Sirius red‐positive areas were quantified separately in the capsule, cortex, and medulla (Figure 2A). The footpad injection and lymphatic vessel ligation predominantly produced linear collagen fibers in the medullary region, whereas the inguinal subcutaneous and intra‐LN injections caused extensive fibrosis in the cortex, with collagen distributed linear and in patches. In contrast, the capsule region showed no obvious differences among the four models (Figure 2B, Figure S2C). Immunohistochemical staining for the fibrosis markers collagen I and α‐SMA showed upregulated expression of both proteins in all four experimental groups compared to the control group (Figure 2C,D). CD45 immunohistochemistry revealed increased immune cell infiltration and elevated CD45 expression in the LNs of all four models, particularly in the subcutaneous and intra‐LN injection groups (Figure 2E).

### 3.3 Evaluation of systemic toxicity in LN fibrosis models

**FIGURE 3:**
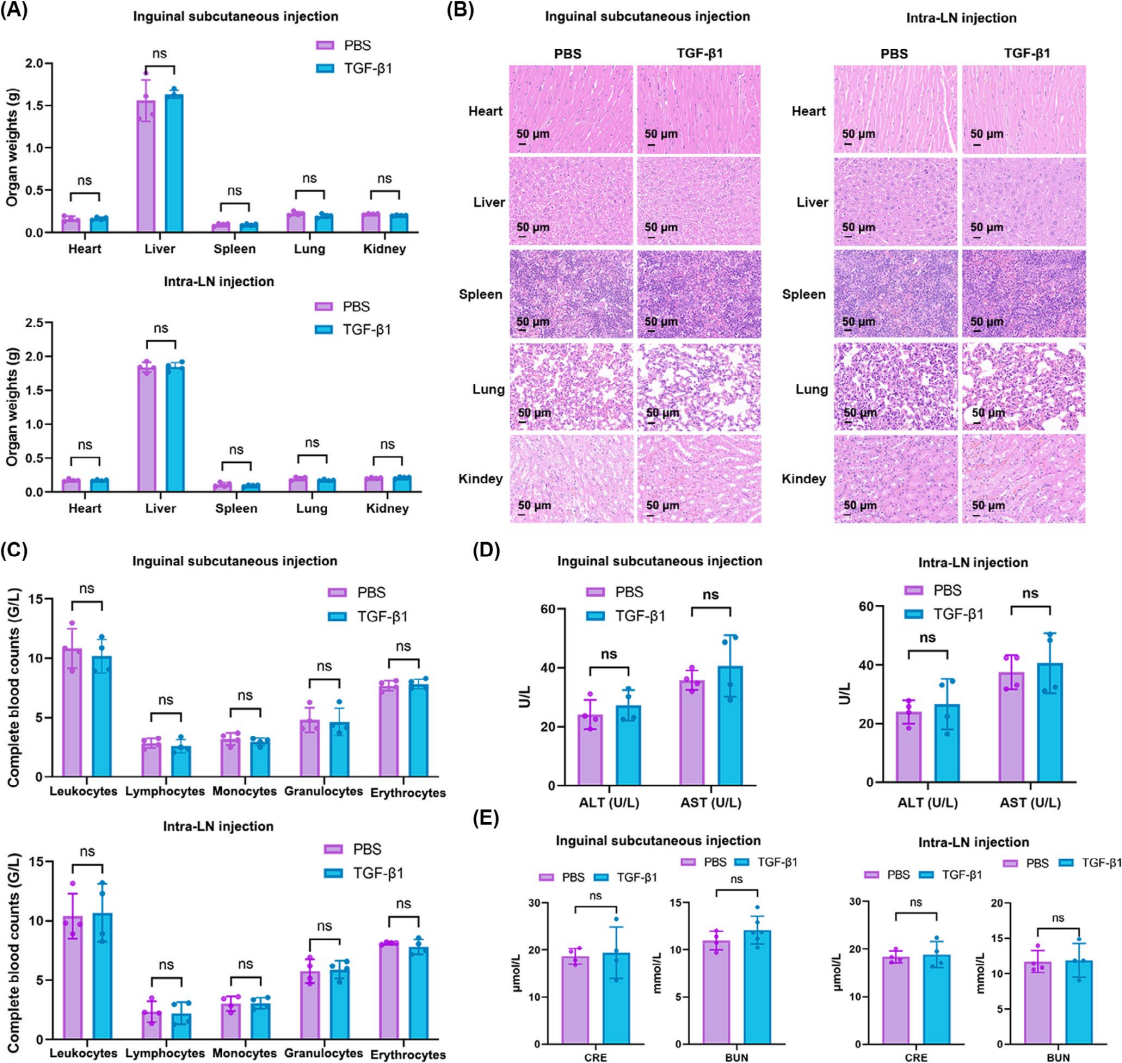
Systemic toxicity assessment of the inguinal subcutaneous and intra‐lymph node (LN) transforming growth factor‐β1 (TGF‐β1) injection models. (A) Organ weights (heart, liver, spleen, lung, kidney) from mice subjected to inguinal subcutaneous or intra‐LN injection were measured to evaluate systemic effects (*n* = 4). (B) Representative hematoxylin–eosin (HE) staining images of major organs from both models (scale bars: 50 μm, *n* = 4). (C) Complete blood cell analysis, including lymphocytes, leukocytes, monocytes, granulocytes, and erythrocytes, comparing the two models with their respective control groups (*n* = 4). (D) Serum alanine aminotransferase (ALT) and aspartate aminotransferase (AST) levels to assess hepatic function (*n* = 4). (E) Serum creatinine (CRE) and blood urea nitrogen (BUN) levels to evaluate renal function (*n* = 4). Data are presented as mean ± standard error of the mean (SEM). ns, not significant.

Based on organ HE staining, complete blood count (CBC) and serum biochemistry for liver and kidney function, both the inguinal subcutaneous and intra‐LN injection models demonstrated in vivo safety and specificity. No significant alterations were observed in the weights of major organs such as the heart, liver, spleen, lung, and kidney (Figure 3A). In HE staining of these organs, none of the groups showed tissue damage, fibrosis, or inflammatory infiltration (Figure 3B). Mice in the LN fibrosis groups did not show any significant differences in the proportions of white blood cells, lymphocytes, monocytes, granulocytes, and erythrocytes (Figure 3C). Moreover, serum biochemical tests showed that liver function indexes (AST and ALT; Figure 3D) and kidney function indexes (BUN and CRE; Figure 3E) were all at normal levels. In summary, these results demonstrated that TGF‐β1–induced LN fibrosis is well tolerated in vivo and does not cause detectable systemic toxicity or organ injury.

### 3.4 Identification of pathways deregulated in fibrotic LN

**FIGURE 4:**
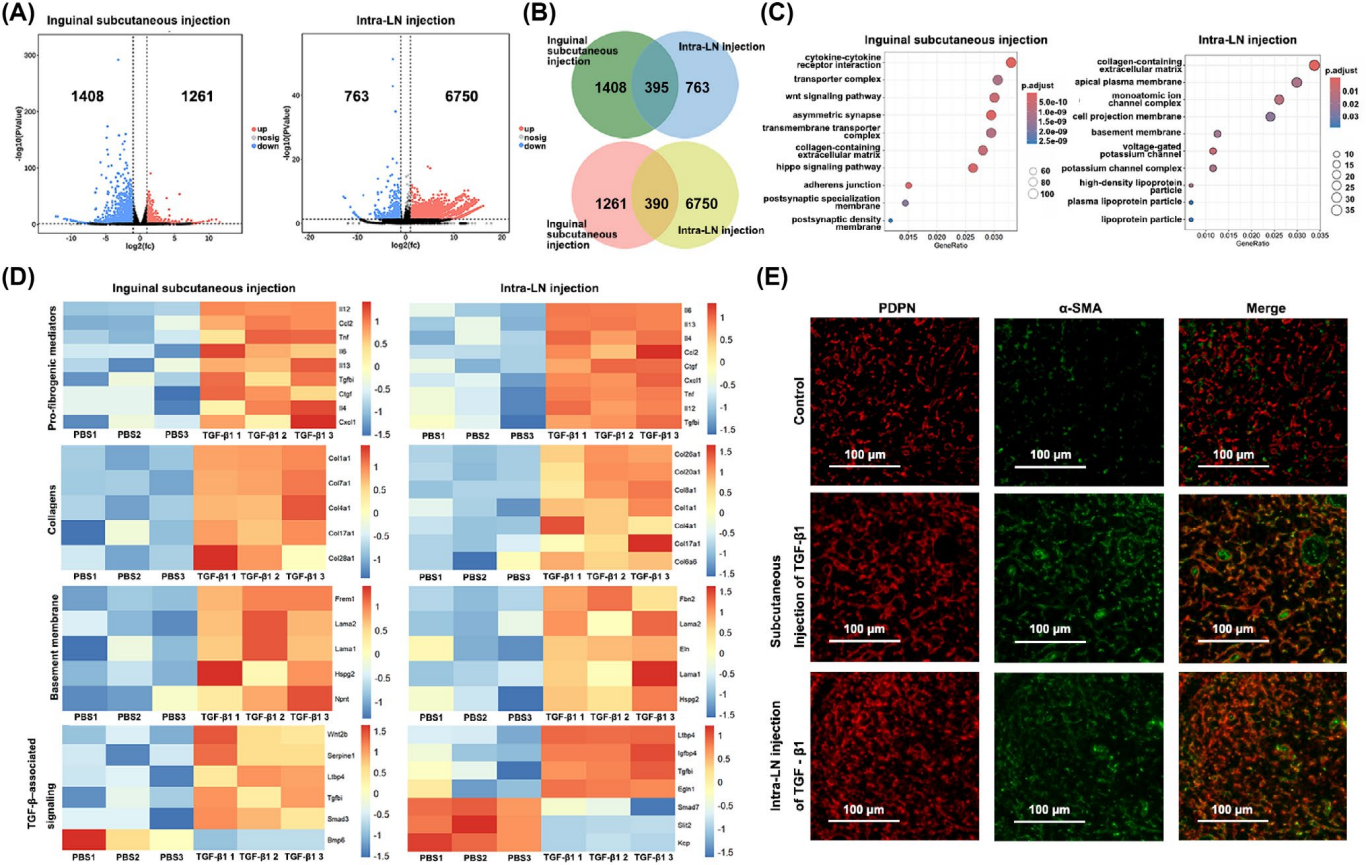
Transcriptomic analysis identifies fibrosis‐related pathways in fibrotic lymph nodes (LNs). (A) Volcano plots showing differentially expressed genes (DEGs) in the inguinal subcutaneous and intra‐LN injection models compared to their respective control groups (*n* = 3; two‐tailed *t*‐test). Significantly downregulated (blue) and upregulated (red) genes are indicated. (B) Venn diagrams showing shared and unique DEGs identified in the two fibrosis models relative to their respective controls. (C) Gene ontology (GO) enrichment analysis of DEGs identified in the inguinal subcutaneous injection and intra‐LN injection models compared to their respective controls. (D) Heatmaps showed gene expression in control and fibrotic LNs, including pro‐fibrogenic molecules, collagens, basement membrane, and transforming growth factor‐β (TGF‐β)–associated signaling pathways. (E) Representative immunofluorescence staining of Podoplanin (PDPN) (red) and α‐smooth muscle Actin (α‐SMA) (green) in control and fibrotic LNs. Scale bars: 100 μm.

Significant differences in gene expression were observed in both the subcutaneous and intra‐LN injection groups (Figure 4A). Specifically, 1408 genes were downregulated in the subcutaneous injection groups, whereas 763 genes were downregulated in the control groups, with 395 genes commonly downregulated under both conditions. However, in contrast to the 1261 genes that were upregulated in the subcutaneous injection groups, 6750 genes were upregulated in the control group, with 390 genes commonly upregulated in both groups (Figure 4B). To investigate the changes in key signaling pathways resulting from these two strategies, we employed pathway enrichment analysis. Gene ontology (GO) enrichment analysis showed that subcutaneous TGF‐β1 injection significantly activated inflammatory response‐related pathways, and the “cytokine–cytokine receptor interaction” was the most enriched term. Intra‐LN injection of TGF‐β1 significantly activated ECM remodeling‐related pathways, with “collagen‐containing extracellular matrix” being the most enriched pathway (Figure 4C). Gene heatmap analysis showed that genes encoding cytokine signaling pathways (such as *Fign*, *Ccl7*, *Vav1*, *Cxcl14*, and *Tgfbr2*) were significantly upregulated after subcutaneous injection of TGF‐β1. Genes encoding ECM‐related molecules, including *Slpi*, *Mfap5*, *Serpine*, *Cxcl14*, were significantly upregulated after intra‐LN injection of TGF‐β1 (Figure S3A,B). To further characterize fibrosis‐associated molecular alterations, DEGs were reorganized into key fibrosis‐related functional groups, including pro‐fibrogenic molecules, collagens, and basement membrane‐related pathways. Genes related to pro‐fibrogenic responses, including *Il12*, *Il13*, *Tnf*, *Cxcl1*, and *Ctgf*, were markedly increased in fibrotic LNs. In addition, multiple ECM remodeling‐associated genes, including multiple collagen family members (*Col1a1*, *Col4a1*, *Col17a1*) and basement membrane‐associated genes (*Lama1*, *Lama2*, and *Hspg2*), were consistently upregulated in both fibrosis models. Elevated levels of genes associated with downstream TGF‐β signaling (such as *Tgfbi* and *Igfbp4*) were observed in fibrotic LNs, supporting the activation of pro‐fibrotic signaling pathways (Figure 4D). Podoplanin (PDPN), a marked of FRCs, largely colocalized with α‐SMA in fibrotic LNs. Compared to control LNs, both fibrosis models showed denser PDPN^+^ FRC networks accompanied by increased α‐SMA expression, indicating stromal activation and structural remodeling following TGF‐β1 treatment (Figure 4E). These findings were consistent with the enrichment of ECM remodeling‐related pathways revealed by GO analysis. In addition, quantitative PCR (qPCR) analysis confirmed significantly increased expression of *Col1a1* and *Acta2* in both fibrotic groups compared to their respective control groups (Figure S3C).

### 3.5 LN fibrosis reduces CD8 ^+^ T cells and increases macrophage infiltration

**FIGURE 5:**
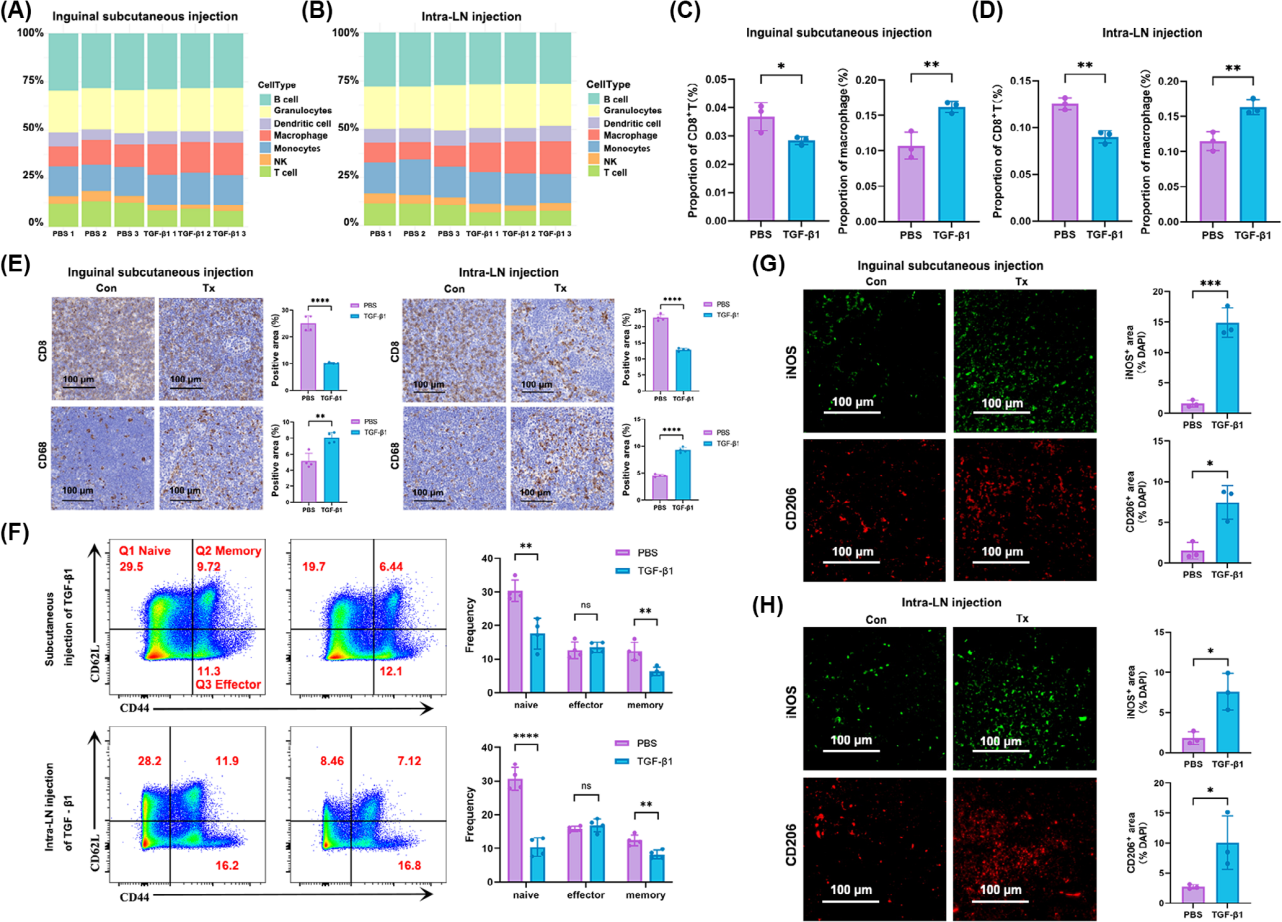
Immune landscape remodeling in fibrotic lymph nodes. (A, B) Stacked bar charts showing the distribution of major immune cell subsets in the inguinal subcutaneous injection model and intra‐lymph node (LN) injection model, alongside their respective controls. (C, D) Proportional quantification of T cells and macrophages across both fibrotic models and their corresponding control groups. (E) Representative immunohistochemical staining and quantification of CD8^+^ T cells and CD68^+^ macrophages in both fibrotic models and controls (scale bars: 100 μm, *n* = 6 random microscopic fields of sections from four mice). (F) Flow cytometric analysis of proportion of CD8^+^ T‐cell subsets in control and fibrotic LNs, including naive (Q1: CD62L^+^CD44^−^), memory (Q2: CD62L^+^CD44^+^), and effector (Q3: CD62L^−^CD44^+^) populations (*n* = 4). (G, H) Representative immunofluorescence staining and quantification of iNOS (green) and CD206 (red) in control and fibrotic LNs from the inguinal subcutaneous injection and intra‐LN injection models (scale bars: 100 μm, *n* = 6 random microscopic fields of sections from three mice). Data are presented as mean ± standard error of the mean (SEM). *p < 0.05, **p < 0.01, ***p < 0.001, ****p < 0.0001; ns, not significant.

To compare changes in immune cell distribution between LN fibrosis and the control group, we used deconvolution analysis to compare immune cell subsets of the two strategies relative to their respective control groups (Figure 5A,B). TGF‐β1–induced fibrosis models have shown a marked reduction in the number of CD8^+^ T cells with upregulated macrophages; it is possible that macrophages could be one key factor contributing to LN fibrotic remodeling (Figure 5C,D; *p* < 0.05). To further confirm these discoveries, we did immunohistochemical staining for CD8 and CD68 in LN sections. Consistent with the transcriptomic and deconvolution data, there was much less infiltration of CD8^+^ T cells in both fibrosis models, and CD68^+^ macrophages accumulated extensively in both fibrosis models (Figure 5E). These results indicate broad immune remodeling in fibrotic LNs, characterized by macrophage enrichment and loss of CD8^+^ T cells.

Flow cytometric analysis further demonstrated alterations in CD8^+^ T‐cell subsets within fibrotic LNs. Compared to control LNs, the proportions of naive (CD62L^+^CD44^−^) CD8^+^ T cells and memory (CD62L^+^CD44^+^) CD8^+^ T cells were significantly reduced in both fibrosis models, whereas the proportion of effector (CD62L^−^CD44^+^) CD8^+^ T cells showed no significant change (Figure 5F). To confirm whether the fibrosis was associated with macrophage polarization, immunofluorescence staining for iNOS and CD206 was performed. Analysis of macrophage phenotypes revealed a marked increase in iNOS^+^ M1 and CD206^+^ M2‐like macrophages in fibrotic LNs (Figure 5G,H).

### 3.6 LOXi attenuates TGF‐β1–induced fibrotic remodeling in LNs

**FIGURE 6:**
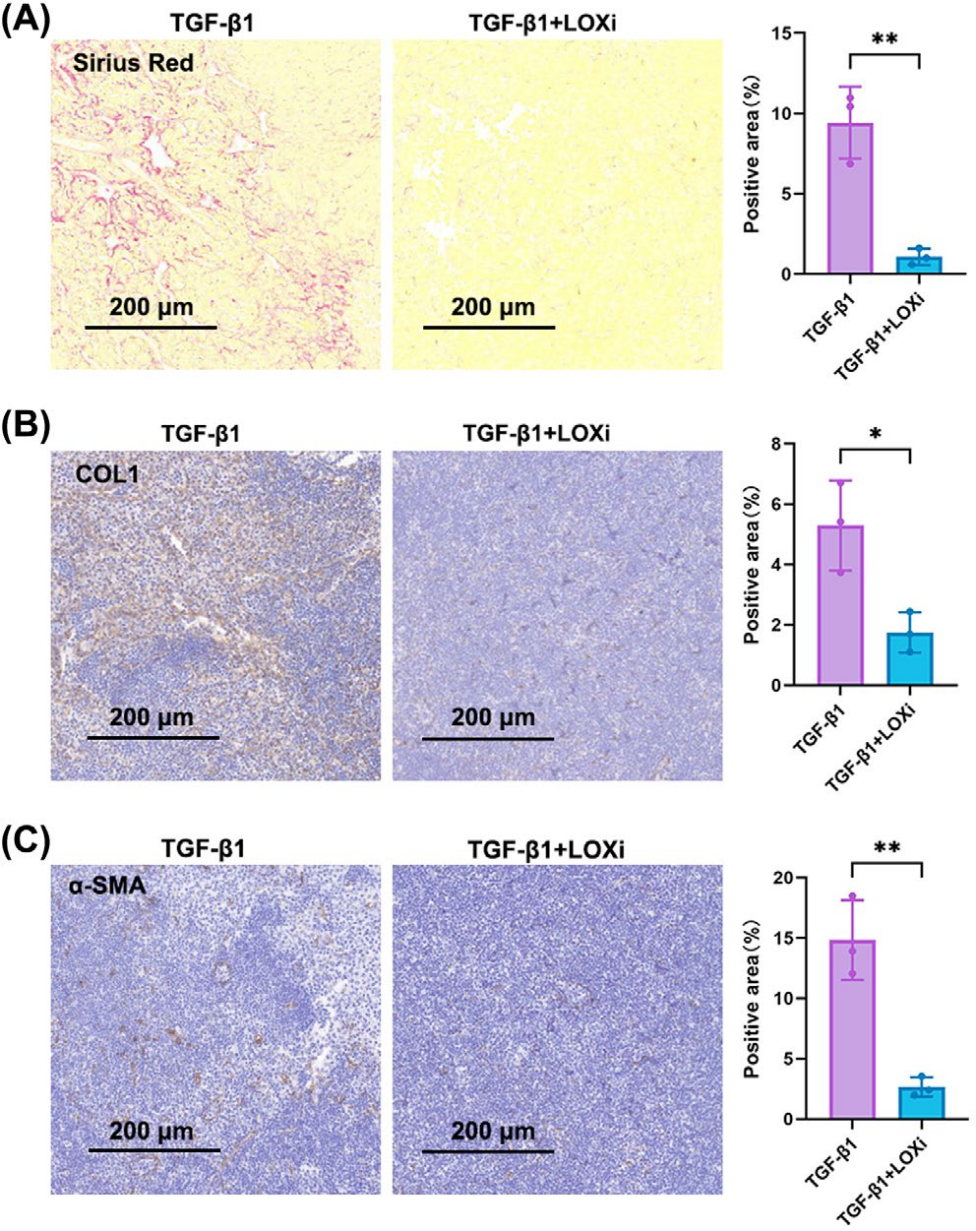
Antifibrotic treatment attenuates transforming growth factor‐β1 (TGF‐β1)–induced lymph node (LN) fibrosis. (A) Representative Sirius red staining and quantitative analysis of collagen deposition in fibrotic LNs induced by intra‐LN injection of TGF‐β1, with or without a lysyl oxidase inhibitor (LOXi) treatment (scale bars: 200 μm, *n* = 6 random microscopic fields of sections from three mice). (B, C) Representative immunohistochemical staining and quantification of the fibrosis‐associated markers COL1 and α‐smooth muscle Actin (α‐SMA) in fibrotic LNs with or without LOXi administration (scale bars: 200 μm, *n* = 6 random microscopic fields of sections from three mice). Data are presented as mean ± standard error of the mean (SEM). *p < 0.05, **p < 0.01.

We next investigated how inhibiting LOX‐mediated collagen cross‐linking using BAPN (LOXi) affected the fibrotic phenotype in LNs. Sirius red staining demonstrated that collagen deposition was significantly reduced in the TGF‐β1+LOXi group compared to the TGF‐β1 group (Figure 6A). Consistently, immunohistochemical staining also confirmed reduced expression of the ECM‐related markers COL1 and α‐SMA in LOXi‐treated LNs (Figure 6B,C). These findings indicate that LOX inhibition effectively alleviates TGF‐β1–induced fibrotic remodeling in LNs.

## 4 DISCUSSION

Anatomically, LNs receive lymphatic drainage from the skin, subcutaneous tissue, and muscle via afferent lymphatic vessels, and therefore they function as the immune system's first responders. The tissue‐derived lymph carries proteins, extracellular vesicles, cytokines, and chemokines, and then enters the narrow conduits along the subcapsular sinus.^21^ Therefore, when these factors continuously drain into the LNs, they can drive fibrosis through chronic inflammation, fibroblast activation, and TGF‐β1–mediated signaling.^22^, ^23^ To simulate the clinical process, this study adopted four different approaches to provoke LN fibrosis. Mice received efferent lymphatic ligation, footpad injection, subcutaneous injection, and direct intra‐LN injection of TGF‐β1. We discovered mild collagen deposition and upregulation of COL1 and α‐SMA in LNs after 2 weeks of TGF‐β1 administration through footpad injections. It may be due to the fact that the injected substance has to flow through the popliteal LNs, the ischial LNs, and then the lumbar LNs in a stepwise manner, greatly reducing the effective concentration that reaches the inguinal LNs.^24^ Efferent lymphatic ligation induced mild fibrosis via obstructive lymph stasis, consistent with the edema–fibrosis progression reported in tail ligation models.^25^ By contrast, both subcutaneous injection and intra‐LN TGF‐β1 injection proved to be more effective. These two methods not only induced robust, sheet‐like collagen deposition, greatly increasing the area of COL1 and α‐SMA positive regions, but also triggered a large infiltration of CD45^+^ immune cells in fibrotic areas.

Our findings further support intra‐LN injection as an effective strategy for localized modulation of the LN microenvironment. Intra‐LN delivery of TGF‐β1 induced pronounced fibrotic remodeling in both the cortical and medullary regions of LNs. A study in cancer immunotherapy has shown that intra‐LN delivery enables antigens and adjuvants to directly accumulate within the same LN microenvironment, thereby promoting coordinated activation of antigen‐presenting cells and lymphocytes, with protective effects lasting for more than 100 days after a single treatment.^26^ These findings suggest that the biological effects of TGF‐β1 within LNs are highly dependent on the route and spatial localization of delivery. Subcutaneous injected and intra‐LN injected optimizing model did not lead to pathological changes in the heart, liver, spleen, lung, and kidney. Serum AST, ALT, CRE, and BUN levels were at control levels; thus, we can say that the method was tissue specific and systemically safe. Due to the pharmacokinetic characteristics of TGF‐β1, it is consumed locally in the short term, and its effective concentration decreases rapidly with increasing delivery distance.^27^ Subcutaneously injected small extracellular vesicles primarily accumulated in LNs instead of liver or spleen.^28^ Moreover, recent studies have further highlighted the potential of engineered extracellular vesicles as efficient carriers for localized delivery of TGF‐β1. Using a CRISPR/Cas9 system to overexpress TGF‐β1 in mesenchymal stem cells, TGF‐β1–enriched extracellular vesicles exhibited enhanced wound healing capacity compared to free TGF‐β1 alone, likely due to improved intracellular delivery efficiency.^29^, ^30^

Beyond gross ECM accumulation, fibrotic LNs displayed strikingly altered transcriptional profiles. TGF‐β1 may initiate immune damage, trigger FRC activation, and cytokine release.^31^ Cytokine–cytokine receptor interaction, Wnt signaling pathway, and Hippo signaling pathway were deregulated after 2 weeks of subcutaneous injection of TGF‐β1, which agrees with typical fibrotic characteristics such as fibroblasts activation and ECM deposition.^32^, ^33^ Enrichment of the adherens junction pathway suggested the formation of a dense, fibrotic ECM.^34^ Four weeks after a single intra‐LN TGF‐β1 injection, collagen‐containing ECM, cell projection membrane, and basement membrane pathway enrichment pointed to the deregulated junction properties. The enrichment of voltage‐gated potassium channel complex pathway may be associated with altered mechanical properties in fibrotic LNs, consistent with a previous study showing that enhanced collagen deposition contributes to increased tissue stiffness.^35^ However, several limitations should also be acknowledged. Because TGF‐β1 exerts broad effects on immune and stromal cells, some of the immune and transcriptomic changes observed in our models may result from TGF‐β1–induced inflammation or tissue responses, rather than fibrosis alone. In particular, direct intra‐LN injection may additionally introduce localized mechanical injury, which could contribute to bystander immune activation. Further studies are still needed to better dissect fibrosis‐specific mechanisms from cytokine‐driven inflammation or injection‐associated tissue responses.

During LN fibrosis, CD45^+^ cells were recruited. In a similar context, after retinal damage, circulating CD45^+^ collagen^+^ fibroblasts were rapidly mobilized from the bone marrow and recruited to the retina to exacerbate fibrosis.^36^ The CD45^+^ population likely also contains other leukocyte subsets, such as neutrophils (CD45^+^Ly6G^+^) or macrophages (CD45^+^F4/80^+^).^37^ Key to LN function, macrophages were upregulated and CD8^+^ T cells in LN were decreased. On the one hand, macrophages play complex and dynamic roles during fibrosis progression. Pro‐inflammatory M1 macrophages mainly contribute to inflammation and tissue injury in early‐stage fibrosis, whereas M2 macrophages promote ECM remodeling and fibroblast activation during chronic fibrosis. In liver fibrosis models induced by bile duct ligation or carbon tetrachloride, FGF12^+^ macrophages drive fibrosis progression by recruiting hepatic stellate cells.^38^ Consistent with these observations, we detected increased infiltration of both M1‐ and M2‐like macrophages in fibrotic LNs, suggesting that both inflammatory and pro‐fibrotic macrophage phenotypes may participate in LN fibrosis. On the contrary, a previous study has reported that macrophages within the tumor's environment can suppress T‐cell function through the consumption of arginine in the local area,^39^ implying that macrophage accumulation in fibrotic LNs may contribute to local immune dysfunction. In addition, TGF‐β1–induced changes may disrupt the survival microenvironment of CD8^+^ T cells. Similar observations have been reported in HIV‐infected LNs, in which collagen deposition and disruption of the FRC network were associated with reduced access of T cells to survival signals such as interleukin‐7 (IL‐7), potentially contributing to T‐cell depletion.^40^

**FIGURE 7:**
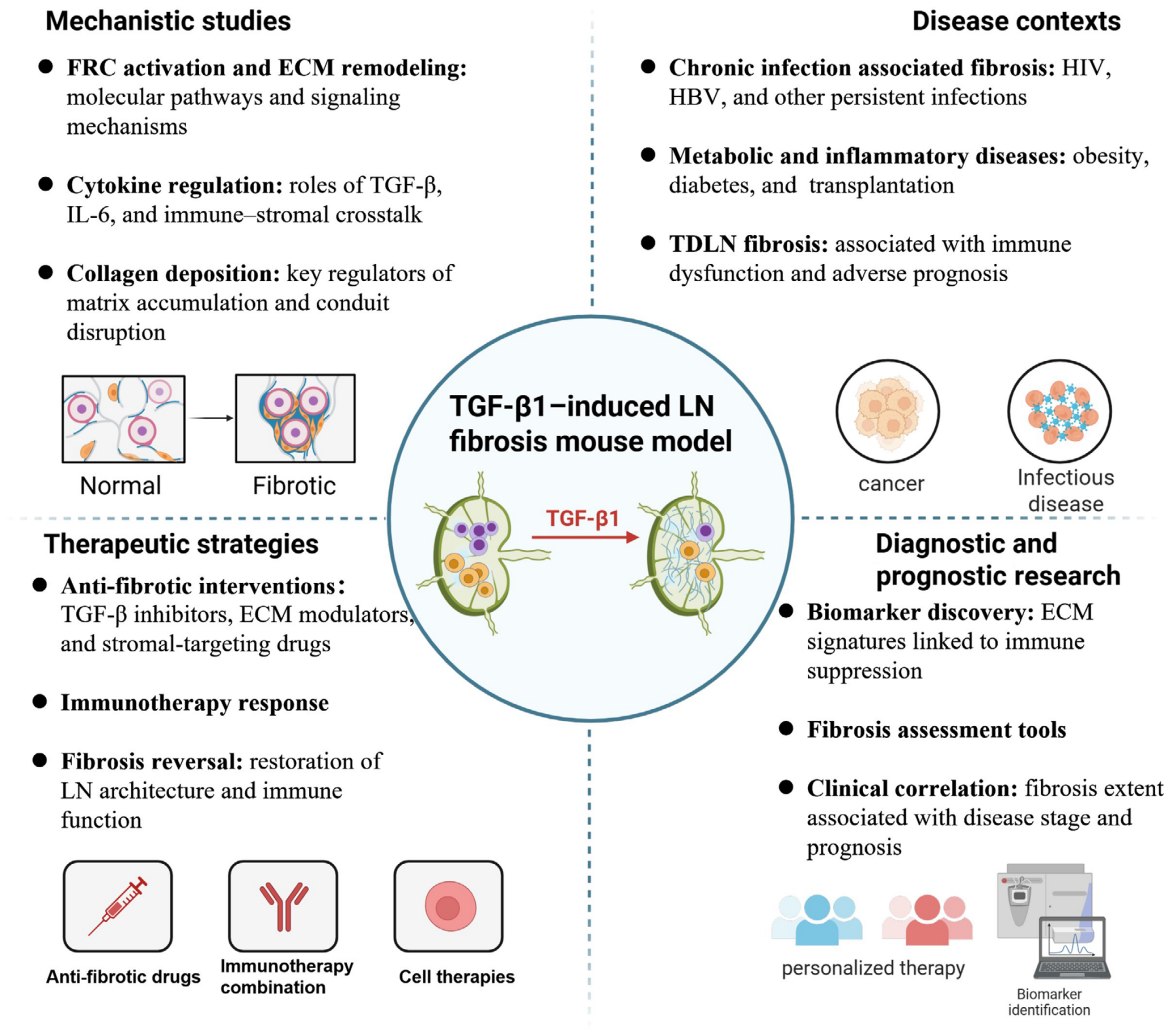
Potential applications and translational value of the lymph node (LN) fibrosis model. This schematic summarizes the multifaceted utility of the model, encompassing (1) mechanistic dissection of stromal–immune interactions; (2) pathophysiological recapitulation across diverse diseases; (3) development of targeted therapeutic strategies, including antifibrotic reversion and immunotherapy sensitization; and (4) identification of diagnostic and prognostic biomarkers based on fibrotic features.

These findings highlight the potential utility of these models for mechanistic discovery, biomarker identification, and translational therapeutic development across a range of disease contexts. Our model provided an experimental platform for decoding how fibrosis‐driven stromal remodeling reshapes immune cell crosstalk within LNs and for identifying potential therapeutic targets. Although existing antifibrotic agents, such as pirfenidone and LOXi, have demonstrated therapeutic benefits by targeting mesenchymal activation and ECM deposition, immune pathway modulation may complement existing antifibrotic strategies. Moreover, recent work further emphasized that the immunosuppressive microenvironment in tumor‐draining lymph node (TDLN) may prevent effective systemic antitumor immune response across multiple cancers.^19^, ^41^ Therefore, antifibrotic strategies should be used in combination with disease‐specific treatments (e.g., antiviral drugs or immune checkpoint inhibitors), rather than applied alone, to achieve better treatment outcomes. Furthermore, high‐throughput omics technologies, including transcriptomics, proteomics, and metabolomics, can more accurately identify biomarkers. Early detection and intervention may help control the dysregulated biological processes that exacerbate fibrosis (Figure 7).

In this study, we developed two new mouse models of LN fibrosis via subcutaneous or intra‐LN TGF‐β1 delivery. Both models produced stronger fibrotic changes than lymphatic ligation or footpad injection without systemic toxicity. The transcriptomic analysis suggested distinct mechanisms between the two models; it is possible because the subcutaneous model is driven by cytokines, whereas the intra‐LN is driven by a mechanical force signal. Both models displayed common features of obvious macrophage infiltration and a decline in CD8^+^ T cells. Therefore, targeting macrophages or their associated pathways holds promise as an effective therapeutic strategy for reversing LN fibrosis. Overall, our study not only offered valuable tools to explore the manifestations of LN fibrosis but also contributed to the development of targeted interventions in future research.

## AUTHOR CONTRIBUTIONS

**Yaru Niu:** Data curation; formal analysis; writing – original draft. **Xu Guan:** Conceptualization; writing – original draft. **Jian Ma:** Formal analysis; methodology; writing – original draft. **Xin Zhang:** Formal analysis; methodology. **Yihang Shi:** Formal analysis; validation. **Jinzhu Zhang:** Methodology; writing – review and editing. **Leiqiong Gao:** Conceptualization; methodology; writing – review and editing. **Wei Chen:** Conceptualization; methodology; supervision. **Xishan Wang:** Funding acquisition; supervision; writing – review and editing.

## CONFLICT OF INTEREST STATEMENT

The authors declare no conflict of interest.

## ETHICS STATEMENT

All animal experiments were approved by the Animal Control Committee of the National Cancer Center/National Clinical Research Center for Cancer/Cancer Hospital of the Chinese Academy of Medical Sciences and Peking Union Medical College (NCC2025A416).

## Supporting information


Figure S1.

Figure S2.

Figure S3.

Data S1.

## Data Availability

The original contributions of this study are included in the article/Supporting Information. For any questions, please contact the corresponding author.
