# Supplementary figures and images for: Establishment and characterization of mouse lymph node fibrosis models
Source: Animal Model Exp Med. 2026 Aug 3:10.1002/ame2.70261. Online ahead of print. doi: 10.1002/ame2.70261 (PMC13430925; doi:10.1002/ame2.70261)

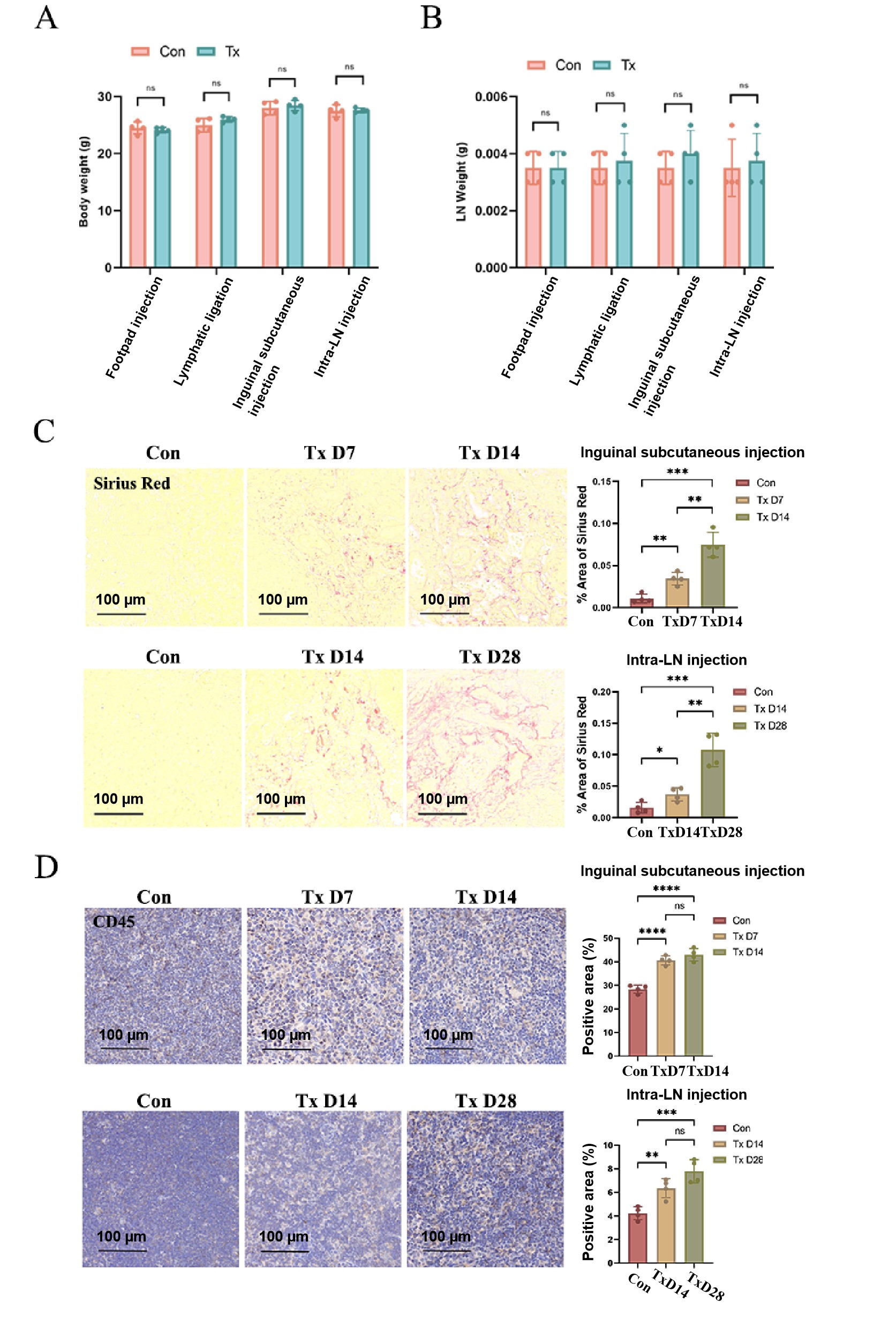

Supplement: Supplementary file 1 — Figure S1. [file AME2-9999-0-s003.jpg]

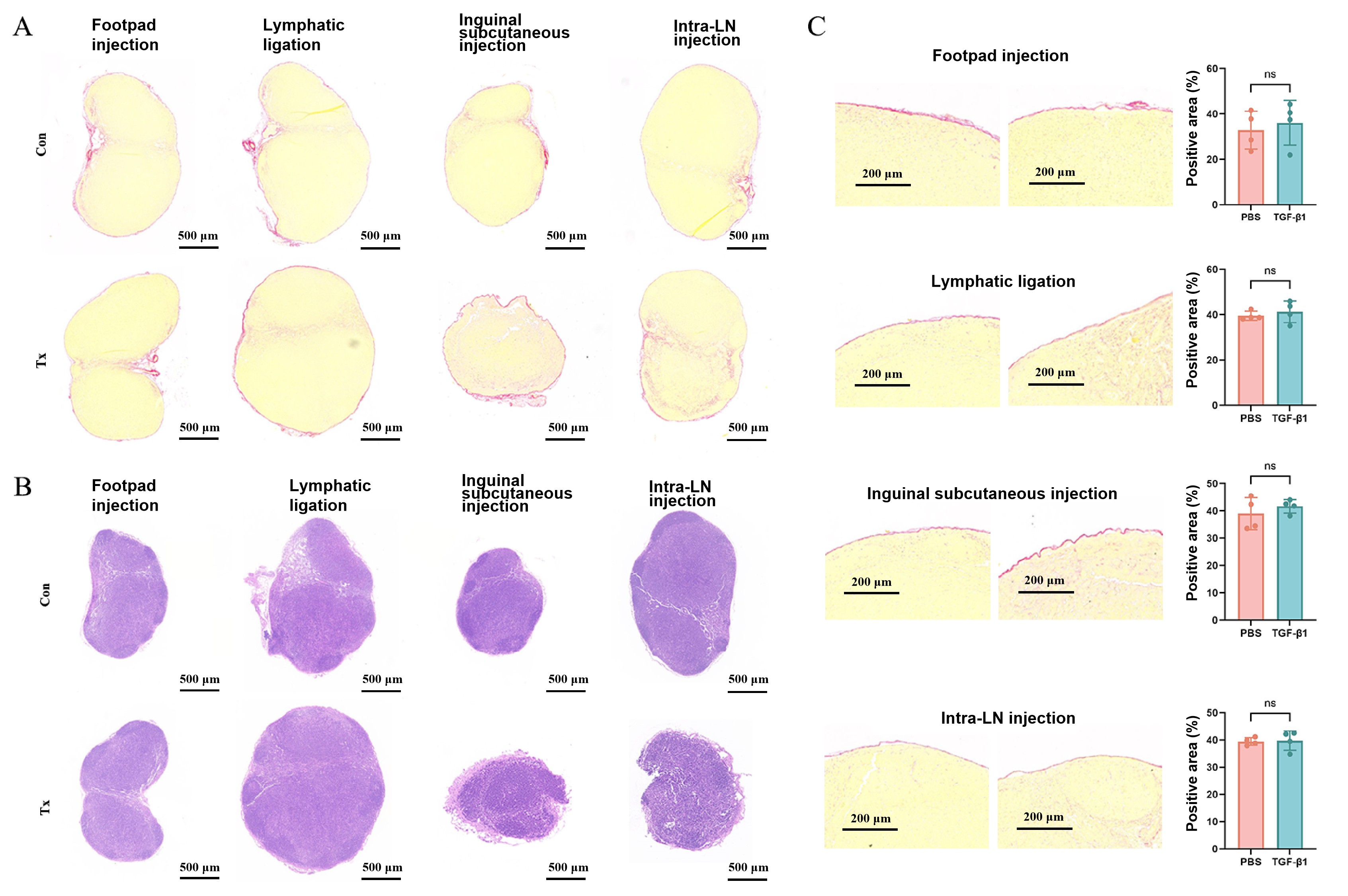

Supplement: Supplementary file 2 — Figure S2. [file AME2-9999-0-s002.jpg]

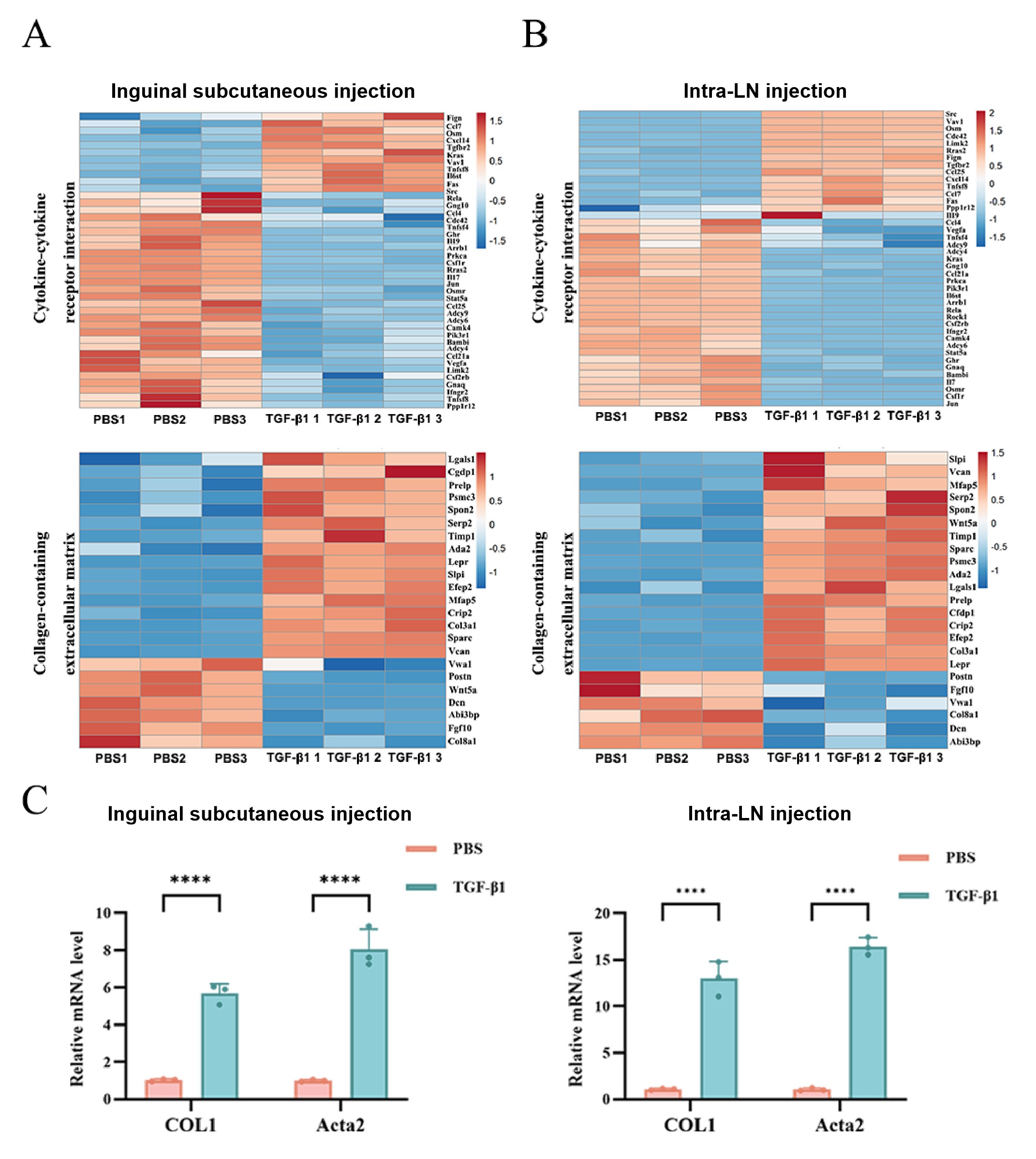

Supplement: Supplementary file 3 — Figure S3. [file AME2-9999-0-s004.jpg]
