# Supplementary material for: Establishment and characterization of mouse lymph node fibrosis models
Source: Animal Model Exp Med. 2026 Aug 3:10.1002/ame2.70261. Online ahead of print. doi: 10.1002/ame2.70261 (PMC13430925; doi:10.1002/ame2.70261)
Supplement: Supplementary file 4 — Data S1. [file AME2-9999-0-s001.docx]

**FIGURE S1** Optimization of the duration of transforming growth factor-β1 (TGF-β1) administration for lymph node fibrosis induction. (A) Body weight of mice in the four modeling groups throughout the experimental timeline (*n* = 4). (B) Lymph node (LN) weights at the experimental endpoint across the four modeling strategies (*n* = 4). (C) Representative Sirius red staining images of LNs (top: inguinal subcutaneous injection; bottom: intranodal injection) and quantitative analysis of collagen deposition at the indicated time points following TGF-β1 administration (scale bars: 100 μm, *n* = 6 random microscopic ﬁelds of sections from four mice). (D) Immunohistochemical staining for CD45 and quantification of the CD45^+^ area in LNs at the indicated time points in both models. Quantification of positive staining areas corresponding to each marker is shown on the right (scale bars: 100 μm, *n* = 6 random microscopic ﬁelds of sections from four mice). Data are presented as mean ± standard error of the mean (SEM). **p* < 0.05, ***p* < 0.005, ****p* < 0.001, *****p* < 0.0001; ns, not significant.

**FIGURE S2** Histological characterization of lymph nodes (LNs) following four fibrosis-induction strategies. (A) Representative Sirius red staining of LNs from the footpad injection, lymphatic ligation, inguinal subcutaneous injection, and intra-LN injection models, along with their respective control groups. Scale bars: 500 μm. (B) Representative hematoxylin–eosin (HE) staining of LNs across the four fibrosis-induction models and their corresponding controls. Scale bars: 500 μm. (C) Representative Sirius red staining and regional quantification of collagen deposition in the capsule of LNs across the four fibrosis-induction models and corresponding control groups (scale bars: 200 μm, *n* = 6 random microscopic ﬁelds of sections from four mice).

**FIGURE S3** Expression of fibrosis-associated genes in fibrotic lymph nodes (LNs). (A,B) Expression patterns of genes from key pathways. Heatmap showed genes related to “cytokine–cytokine receptor interaction” and “collagen-containing extracellular matrix” in both fibrotic models and their controls. (C) Messenger RNA (mRNA) expression levels of COL1 and Acta2 in the inguinal subcutaneous injection model and the intra-LN injection model and their respective controls (*n* = 3). Data are presented as mean ± standard error of the mean (SEM). *****p* < 0.0001.
